# Combination of Fenretinide and Selenite Inhibits Proliferation and Induces Apoptosis in Ovarian Cancer Cells

**DOI:** 10.3390/ijms141121790

**Published:** 2013-11-04

**Authors:** Jie Liu, Jia Li, Jian-Fang Zhang, Xiao-Yan Xin

**Affiliations:** Department of Obstetrics and Gynecology, Xijing Hospital, the Fourth Military Medical University, Xi’an 710032, China; E-Mails: liujie007@fmmu.edu.cn (J. Liu); jiali_mmu@126.com (J. Li); jianfangzhang@126.com (J.-F.Z.)

**Keywords:** ovarian cancers cell, fenretinide, selenite, combination, anti-tumor effect

## Abstract

The combination of fenretinide and selenite on ovarian cancer cells was investigated to assess its effects on proliferation and ability to induce apoptosis. Our results showed that fenretinide and selenite in combination significantly suppress the proliferation of ovarian cancer cells and induced apoptosis (including reactive oxygen species generation, and the loss of mitochondrial membrane potential) compared with either drug used alone. The caspase3/9-dependent pathway was triggered significantly in combination treatment, and moreover, the AMPK pathway also mediated the apoptosis induction in fenretinide and selenite combination. Fenretinide and selenite combination treatment was demonstrated to suppress tumor growth *in vivo*, this drug combination has been thus found to have an enhanced anti-tumor effect on ovarian cancers cells.

## 1. Introduction

Gynecologic cancers such as ovarian cancer is a common type of cancer in women, the prognosis for patients with these advanced cancers is extremely difficult; and the conventional chemotherapy does not have clinical efficiency for advanced ovarian cancer patients [1]. Thus, novel therapeutic strategies are urgently needed.

All-trans retinoic acid (ATRA) was previously widely used for treating acute promyelocytic leukemia (APL) [2–6]. However, in order to reduce the observed retinoid treatment-related side effects such as liver toxicity [7–10], several ATRA analogues have been synthesized. One of them is *N*-(4-hydroxy-phenyl)-retinamide (4-HPR, fenretinide, Figure S1) [11–13]. Holmes *et al.* found that two synthetic retinoids CD437 and 4-HPR initially activate separate pathways to induce mitochondrial depolarization but both utilize mitochondrial depolarization, caspase-9 activation, and caspase-3 activation in the later stages of ovarian tumor cell line apoptosis induction [14]. Other studies have also demonstrated that fenretinide has great potential in cancer chemoprevention and therapy through cell proliferation suppression and apoptosis induction in a variety of human cancer cell types [15–18].

Selenium is an essential trace element existing in organic and inorganic chemical form’s which have been shown to play an important role in the maintenance of an optimal physiological state of mammalian cells. Dietary selenium deficiency is associated with increased risk for heart disease, immune dysfunction, male infertility and cancer [19–21]. And selenium is an essential micronutrient incorporated into proteins in the form of the amino acid selenocysteine [22–24]. In addition, recent reports have shown that selenium has chemo-preventive property against a wide range of malignancies [25]. However, accumulating evidence shown that selenium has cytotoxic effects at high doses [23].

The effects of selenium compounds depend on the drug administration and dose used. At low doses, it has antioxidant with cancer preventing properties; while at a higher dose it has been shown promising tumor specific cytotoxic effect. Clinical trials have shown that selenium is effective for the prevention of cutaneous melanoma [26], and the drug as part of a combined drug treatment may have an enhanced anti-tumor effect on some cancer cells [27]. Therefore, in present study, we examined the potentiating or enhanced effect of fenretinide when combined with selenite in ovarian cancer cells.

## 2. Results

### 2.1. Fenretinide and Selenite Enhance Suppression of Proliferation of Ovarian Cancer Cell Lines

**Figure 1:**
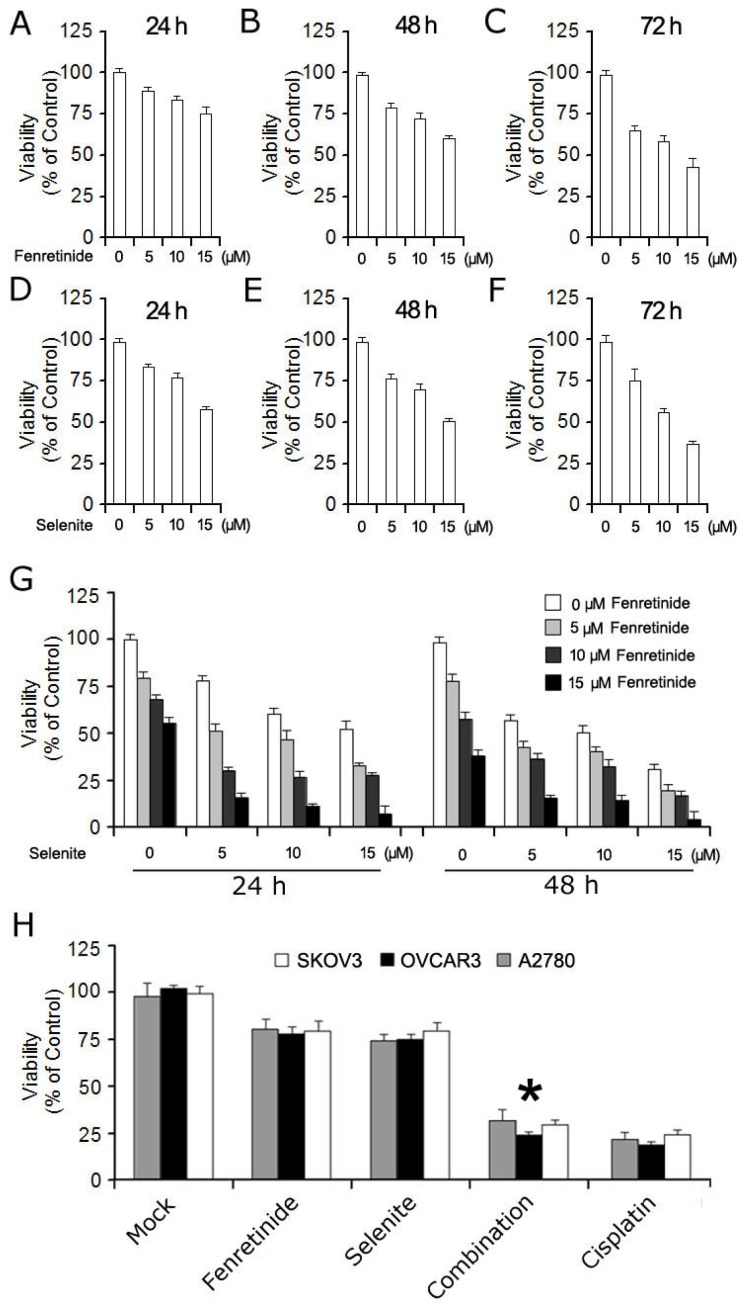
Effect of fenretinide and selenite on ovarian cancer cells proliferation. Viability of SKOV3 cells was measured by MTT after exposure of fenretinide with different concentrations for 24 h (**A**); 48 h (**B**) and 72 h (**C**); Viability of SKOV3 cells measurement after exposure of selenite with different concentration for 24 h (**D**); 48 h (**E**) and 72 h (**F**); SKOV3 cells’ viability under different concentration of fenretinide and selenite combination treated for 24 h and 48 h (**G**); Viability assay using two additional different ovarian cell lines (OVCAR3 and A2780) after exposure of drugs alone (10 μmol/L fenretinide, 10 μmol/L selenite) or drug combination (10 μmol/L fenretinide plus 10 μmol/L selenite) for 48 h, 10 μg/mL cisplatin treatment used as positive control (**H**). (means ± s.d., *p < 0.05).

Fenretinide and selenite were first used separately to treat ovarian cancer cell lines, and then analyzed by MTT assay. As shown in Figure 1, fenretinide (Figure 1A–C) and selenite (Figure 1D–F) both significantly reduced viability of ovarian cancer cell line SKOV3 (*p* < 0.05). The fenretinide (5–15 μmol/L) and selenite (5–15 μmol/L) combination was then assessed for 24 and 48 h; the data shows that the combination of both agents exhibited a potentiating or enhanced effect (*p* < 0.01) (Figure 1G). Moreover, two different additional ovarian cancer cell lines, OVCAR3 and A2780, were also used to test the potentiating or enhanced effect of this combination; the cell viability of two cell lines also significantly decreased (Figure 1H), indicating that the fenretinide and selenite combination could suppress the growth of ovarian cancer cell lines additively.

### 2.2. Fenretinide and Selenite Induce Ovarian Cancer Cell Lines Apoptosis

**Figure 2:**
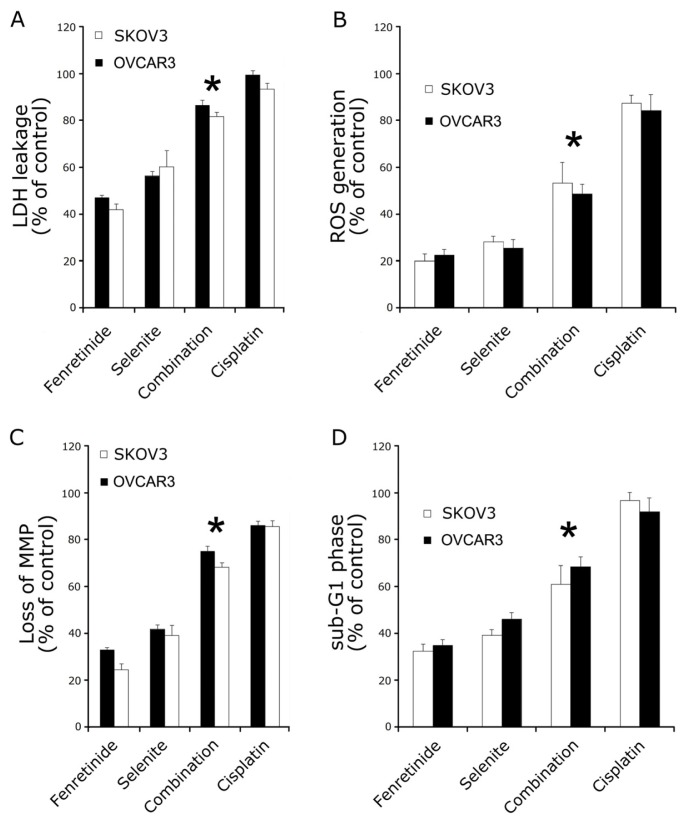
Fenretinide and selenite cytotoxicity on SKOV3 and OVCAR3 cells. After drug alone (10 μmol/L fenretinide, 10 μmol/L selenite) or drug combination (10 μmol/L fenretinide plus 10 μmol/L selenite) exposure for 48 h, lactate dehydrogenase (LDH) leakage assay (**A**); intracellular reactive oxygen species (ROS) generation assay (**B**); mitochondrial membrane potential (MMP) loss assay (**C**) and the sub-G1 phase cells analysis (**D**) were performed. (means ± s.d., *p < 0.05).

**Figure 3:**
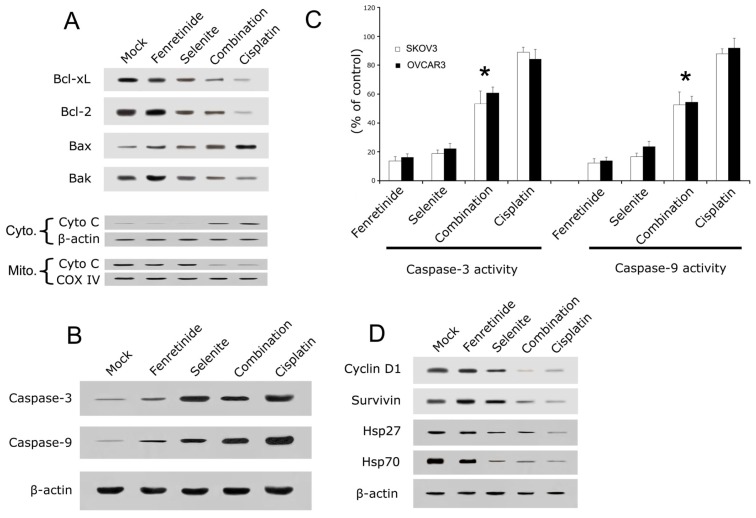
Apoptosis related protein expression by Western blotting and the caspase 3/9 activity analysis. After drug alone (10 μmol/L fenretinide, 10 μmol/L selenite) or drug combination (10 μmol/L fenretinide plus 10 μmol/L selenite) exposure for 48 h, Western blotting was performed with antibodies against apoptosis related protein such as Bcl-xL, Bcl-2, Bax, Bak and cytochrome c (**A**); Cleaved caspase-3 and caspase-9 (**B**); Caspase-3 and Caspase-9 activity was also measured (**C**); Cell cycle related protein Cyclin D1 and Survivin, the heat shock proteins Hsp27 and Hsp70 expression were analyzed (**D**). (means ± s.d., *p < 0.05).

To determine whether the inhibitory effect of fenretinide and selenite on the cell viability is related to the induction of apoptosis, lactate dehydrogenase (LDH) leakage assay, intracellular reactive oxygen species (ROS) assay, mitochondrial membrane potential (MMP) loss assay and flow cytometry analysis were performed. As is shown in Figure 2, 10 μmol/L fenretinide and 10 μmol/L selenite combination were used to treat SKOV3 and OVCAR3 cells. Cytotoxicity of the drug combination was measured by LDH leakage assay, as is shown in Figure 2A. The LDH leakage in drug combination increased significantly compared to drug used alone. ROS was measured as an increase in fluorescence intensity quantification by monitoring the enzymatic cleavage of DCFH-DA expressed as a percentage of untreated control relative to ROS levels. In combination exposure, ROS generation significantly increased relative ROS as observed at 24 h (Figure 2B). MMP loss usually precedes or accompanies with ROS production, and the dye JC-1 was used to monitor the MMP loss after drug exposure. When the cells were exposed to the drug treatment for 24 h, MMP loss was significantly increased (Figure 2C). Lastly, apoptosis assay by flow cytometry was performed after drug treatment; the number of cells in the sub-G1 phase (Figure 2D) was significantly increased when compared to the drugs used alone (*p* < 0.05). Bcl-2, Bcl-xL and Bak expression decreased after combination treatment, while Bax expression increased; the combination induced the release of cytochrome c from mitochondria to the cytosol (Figure 3A).

Apoptotic signals are known to induce the activation of caspase cascades; Holmes *et al.* showed that fenretinide-induced ovarian cancer cell apoptosis requires the activation of caspases [28]. In order to analyze whether the fenretinide and selenite combination can significantly enhance the activation of caspases on ovarian cancer cells apoptosis, caspase-3 and caspase-9 expression and activity were examined. In Figure 3, cleaved caspase-3 and caspase-9 expression was significantly increased after the fenretinide and selenite combination exposure in SKOV3 cells (Figure 3C), and the activity of caspase-3 and caspase-9 (Figure 3B) increased significantly with the drug combination, which indicated that the activation of caspases is associated with the apoptotic process in SKOV3 and OVCAR3 cells. Cell cycle or proliferation related proteins such as Cyclin D1, Survivin, Hsp 27 and Hsp 70 decreased significantly with the drug combination treatment (Figure 3D).

### 2.3. AMPK Mediated Fenretinide and Selenite Combination-Induced Apoptosis

**Figure 4:**
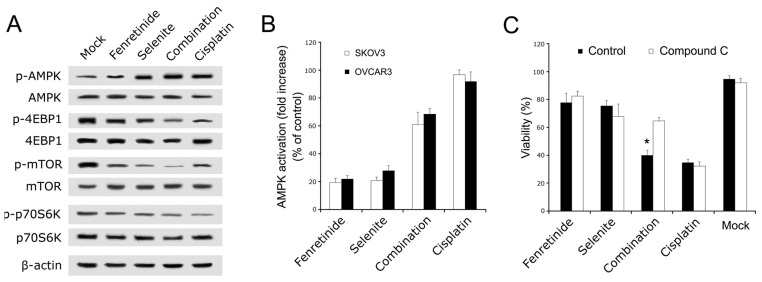
AMPK signaling pathway assay. After drug alone (10 μmol/L fenretinide, 10 μmol/L selenite) or drug combination (10 μmol/L fenretinide plus 10 μmol/L selenite) exposure for 48 h, Western blotting analysis for the expression of phosphorylated AMPK, 4EBP1, p70S6K and mTOR from AMPK signaling pathway (**A**); Cell-based enzyme-linked immunosorbent analysis for intracellular concentrations of phospho-AMPK of SKOV3 and OVCAR3 cells (**B**); Viability of SKOV3 cells under compound C (AMPK inhibitor) treatment (**C**). (means ± s.d., *p < 0.05).

To further examine the processes before apoptosis induction by fenretinide and selenite combination, we analyzed the AMPK signaling pathway (AMPK pathway down stream signaling molecules including AMPK, mTOR, p70S6K, and 4EBP1 [29]). The phosphorylation status of AMPK, mTOR, p70S6K, and 4EBP1 proteins after drug combination treatment were assessed by Western blotting. Phosphorylation of AMPK increased, while there was decreased phosphorylation of mTOR, P70S6K, and 4EBP1 (Figure 4A). Moreover, data from cell-based ELISA have shown that AMPK phosphorylation significantly increased after drug combination exposure of SKOV3 and OVCAR3 cells (Figure 4B). In order to analyze whether the potentiating or enhanced effect of the combination is mediated by the AMPK pathway, AMPK signaling pathway was blocked by the AMPK inhibitor compound C. SKOV3 cells viability were rescued by compound C (5 mM) after drug combination (Figure 4C). Therefore, the AMPK signaling pathway may be necessary to induce ovarian cancer cell apoptosis after combination treatment with fenretinide and selenite.

### 2.4. Fenretinide and Selenite Combination on Tumor Growth in SKOV3 Xenograft Model

**Figure 5:**
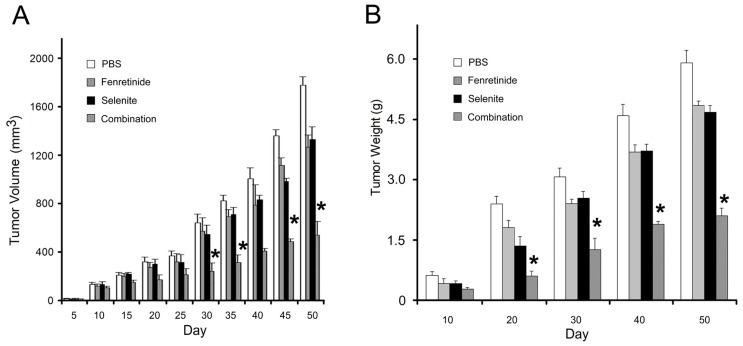
*In vivo* anti-tumor activity of fenretinide and selenite against SKOV3 cells-bearing model. Tumor volumes were measured every 5 days after initiating single drug or drug combination therapy (**A**); Tumor weight of tumor bearing mice received the drug alone or drug combination therapy was measured every 10 days (**B**). SKOV3 cells-bearing model treated with PBS were used as control (means ± s.d., *p < 0.05).

In order to examine the effect of fenretinide and selenite combination on tumor growth *in vivo* (*in vivo* experiment protocol was followed as described [27]), we used a xenograft nude mouse tumor model with subcutaneously implanted SKOV3 cells. Fifty days after the start of treatment, the tumor volume was measured every five days, and the tumor weight was measured every ten days. In the combination group, the tumors grew slowly in volume (Figure 5A) and weight (Figure 5B), while, in the single drug group, the tumors grew faster than in the combination group. The fenretinide and selenite combination could therefore significantly decrease the tumor growth *in vivo*, demonstrating the synergistic effect of these two drugs in combination.

## 3. Discussion

Although much effort has been made in anti-ovarian cancer therapy in recent years, there are still no effective low-toxicity drugs for treating ovarian cancer. Therefore, potent anti-ovarian cancer drugs or newly therapeutic methods are highly desired. Fenretinide, a synthetic derivative of retinoic acid, could induce apoptosis in a wide range of cancer cells *in vitro* [3–6], Holmes *et al.* identified the signaling pathways of apoptosis induced by fenretinide treatment on ovarian carcinoma cells [28]. Moreover, selenite is an essential trace element for the body to synthesize selenoproteins [30–33], and some studies shown that selenite within the nutritional range concentration can inhibit tumor formation [34–38], but in the over nutritional levels, selenite can induce endoplasmic reticulum stress, mitochondrial related apoptosis, DNA strand breaks and cell-cycle arrest [39–41], which suggests that selenite has potential therapeutic effects for cancer treatment [42–45].

According to some previous reports, drug combinations for cancer treatment often provide a promising approach [15,17]. Additionally, combined treatment of NB4 cells with ATRA at low concentration and sodium selenite exhibited a synergistic effect on apoptosis induction [46]. However, the anti-tumor effect of these two drugs using separate treatment on cancer cells is limited.

Combination chemotherapy could optimize the effectiveness of each drug by inciting a complimentary and synergetic therapeutic response while concurrently reducing side effects associated with single-agent therapy. Thus, we investigated whether a combination treatment with fenretinide and selenite on ovarian cancer cells has a synergistic effect. Here, we show the viability of ovarian cancer cell lines was significantly decreased by fenretinide and selenite combination treatment. In addition, ovarian cancer cell lines with the combination treatment were also demonstrated to have increased levels of induced apoptosis compared to single drug treatment.

There is growing evidence that some cell metabolism related signaling pathways drive cancer cell growth [47]. AMPK is activated by the depletion in cellular energy levels, and allows adaptive changes in cell metabolism and cell survival [29], and recent reports have shown that the AMPK family member Snf1 protects Saccharomyces cerevisiae cells upon glutathione oxidation [48], moreover, reactive oxygen species play a role in cell apoptosis. Based on these cues, we investigated whether the AMPK pathway is involved in the fenretinide and selenite combination treatment. Analysis of the AMPK pathway by Western blotting, AMPK activities and the AMPK inhibitor assay, demonstrated that the AMPK pathway plays a key role in the fenretinide and selenite combination treatment. Therefore, the AMPK signaling pathway may play an important role in growth suppression and apoptosis induction in fenretinide and selenite combination. Lastly, *in vivo* studies, using tumor growth in mice revealed a decrease in tumor growth by the combination therapy.

In summary, fenretinide and selenite combination treatment on ovarian cancer cell proliferation suppression and apoptosis have a synergistic effect, and may involve the AMPK-dependent pathway. Our data provide a novel insight into the potential application of fenretinide and selenite for ovarian cancer, and provides support for further clinical evaluation of the combination of fenretinide and selenite for ovarian cancer patients.

## 4. Materials and Methods

### 4.1. Cell Lines, Cell Culture and Reagents

Human ovarian cancer SKOV3, OVCAR3 and A2780 cell lines were purchased from American Type Culture Collection (ATCC, Manassas, VA, USA). These cell lines were cultured in DMEM medium (Sigma, St. Louis, MO, USA) supplemented with 10% heat-inactivated fetal bovine serum (FBS, Hyclone, Logan, UT, USA), 100 U/mL penicillin and 100 U/mL streptomycin. Cultures were incubated in a humidified atmosphere containing 5% CO_2_ at 37 °C.

Drugs and kit were obtained as follow: Sodium selenite and Fenretinide (Sigma Chemicals, St. Louis, MO, USA); JC-1 Mitochondrial Membrane Potential Detection Kit (Abcam, Cambridge, MA, USA); Selenite was dissolved in distilled water to the final stock concentration of 1 mM, while fenretinide was dissolved in DMSO (final stock concentration of 1 mM). Working concentration obtained by diluting the stock solution with treatment medium.

Antibodies were purchased as follow: anti-β-actin, anti-Bcl-2, anti-Bax, anti-Bcl-xL, anti-Bak, anti-Survivin, anti-Cyclin D1, anti-Hsp27/70, anti-Cyto C, anti-P-AMPK, anti-AMPK, anti-p-p70S6K, anti-p70S6K, anti-p-mTOR, anti-mTOR, anti-4EBP1, anti-p-4EBP1, and anti-caspase-3/9 (Catalog #9661/#9501) were purchased from Cell Signaling Technology (Boston, MA, USA). Horseradish peroxidase-conjugated anti-rabbit IgG, anti-mouse IgG, and enhanced chemiluminescene (ECL) reagents were obtained from Pharmacia-Amersham (Amersham, UK).

### 4.2. Cell Viability Assay

Cell proliferation ability was detected by the MTT analysis (Sigma, St. Louis, MO, USA). Firstly, cells were seeded in 96-well plates at a density of 5 × 10^3^ cells per well, incubated for 24 h. To determine the growth inhibitory effect of fenretinide, selenium and their combinations on SKOV3, OVCAR3 and A2780 cells. Before the drug treatment, the culture medium changed as 2% FBS in RPMI 1640 medium (Gibco, Gran Island, NY, USA). After 24, 48 and 72 h treatment with varying doses of single drug or its combination. On the day of collection, 200 μL of MTT solution was added to the well and the cells were incubated at 37 °C for 4 h. The MTT-containing medium was removed and DMSO (200 μL) was added for 30 min to dissolve MTT formazan crystals and the absorbance at 570 nm was measured using a multiwell plate reader (BioTek, Winooski, VT, USA). Wells containing only RPMI1640 and MTT were used as mock. Cell viability was calculated as percentage of viable cells in total population. Each experiment was performed with three replicates.

### 4.3. Lactate Dehydrogenase (LDH) Leakage Assay

The release of cytoplasmic lactate dehydrogenase (LDH) into the culture medium was determined following the protocol described [49]. After ovarian cancer SKOV3 and OVCAR3 cells treated with drugs alone or combination for 24 h, the culture medium was aspirated and centrifuged at 2000 × *g* for 10 min to obtain a cell free supernatant. LDH activity in medium was examined by conversion of lactate to pyruvate using a commercially available kit (Sigma, St. Louis, MO, USA).

### 4.4. Intracellular Reactive Oxygen Species (ROS) Measurement

Generation of ROS was assessed using DCFH-DA dye as a fluorescence agent. Briefly, SKOV3 and OVCAR3 cells (1 × 10^4^ per well) were seeded in 96-well black bottom culture plate and allowed to adhere for 24 h in a CO_2_ incubator at 37 °C. The cells were then challenged with fenretinide and sodium selenite for 24 h. The medium was discarded and the cells were incubated with DCFH-DA (10 μM, *E*_x_/*E*_m_ = 485 nm/528 nm) for 30 min at 37 °C in the dark. The reaction mixture was aspirated and replaced by 200 μL of PBS in each well. The plates were kept on a shaker for 10 min at room temperature in the dark. Fluorescence intensity was measured using a Multiwell microplate reader (FLUOstar, Durham, NC, USA), and the values were expressed as a percentage of fluorescence intensity relative to the control wells.

### 4.5. Propidium Iodide (PI) Staining and Flow Cytometry Analysis

Sub-G1 distribution were determined by the staining of DNA with PI (*E*_x_/*E*_m_ = 488 nm/617 nm). In brief, 1 × 10^6^ cells were incubated with single or dual agents for 24 h. On the day of collection, the cells were harvested and washed twice with ice-cold PBS. The cells were fixed with 70% ice-cold ethanol at 4 °C for 1 h. The cells were washed once with PBS and resuspended in a staining solution containing PI (50 mg/mL) and RNase A (250 mg/mL). The cell suspensions were incubated for 30 min at room temperature followed by flow cytometry (Beckman Coulter, Fullerton, CA, USA) using 20,000 cells for each group.

### 4.6. Mitochondrial Membrane Potential Assays

Apoptosis often causes the mitochondrial membrane potential (MMP) loss, and this MMP loss could be detected by JC-1 (*E*_x_/*E*_m_ = 525 nm/610 nm). SKOV3 and OVCAR3 cells were treated with fenretinide and sodium selenite, or its combination for 24 h, harvested in 5 mL polystyrene tubes, and then centrifuged at 400 × *g* for 5 min; the cell pellet was resuspended in 10 μg/mL of JC-1, incubated at 37 °C for 20 min. Cells were washed and resuspended in PBS, and then fixed with 4% paraformaldehyde. After another wash in PBS, the cells on the microplate were read by a spectrophotometer.

### 4.7. Assay of Caspase Activity

The assay is based on the cleavage of the chromogenic substrates, DEVD-pNA and LEHD-pNA, by caspase-3 and caspase-9, respectively. The activity of the caspase-3 and caspase-9 was determined using a Diagnostic Reagent kit (Nanjing Jiancheng, Nanjing, China) according to the manufacturer’s protocol. Briefly, both treated and untreated SKOV3 and OVCAR3 cells were washed with PBS, and then the cells were harvested with lysis buffer on an ice bath. The cell lysate were centrifuged at 10,000 × *g* for 10 min, and 200 μg of proteins was incubated with appropriate protease assay buffer and appropriate substrate, respectively, at 37 °C for 4 h. The optical density of the reaction mixture was detected by a spectrophotometer (Molecular Devices, Sunnyvale, CA, USA) at the wavelength of 405 nm. Experiments were performed at least three times.

### 4.8. AMPK Activation Assay

Cell-based enzyme-linked immunosorbent assay (ELISA) [50] was used to detect the AMPK activity. After treatment, cells were fixed in 4% PFA; 1% H_2_O_2_ in PBS containing 0.1% Triton X-100 (Sigma) was used to quench endogenous peroxidase; blocked with 10% FBS. Primary specific rabbit polyclonal antibody for mouse phospho-AMPK was incubated at 37 °C for 1 h, then the secondary peroxidase-conjugated goat anti-rabbit IgG undertaken for another 1 h. After incubation with the peroxidase substrate tetramethylbenzidine, the reaction was stopped with HCl. The absorbance of the cells on the microplate was measured by a spectrophotometer at 450 nm. The obtained absorbance was corrected for the cell number determined by crystal violet staining [51]. Experiments were performed at least three times.

### 4.9. Subcellular Fraction Isolation

After treatment of fenretinide and sodium selenite, cells were collected by centrifugation at 1000 × *g* for 5 min. Cell pellets were resuspended with 5.5 mL of cold RSB buffer (10 mM NaCl, 1.5 mM MgCl_2_, 10 mM Tris-HCl, pH 7.5, supplemented with protease and phosphatase inhibitors) and incubated on ice for 90 min. Cells were then lysed in a dounce homogenizer and mixed with 4 mL 2.5 × MSB buffer (525 mM Mannitol, 175 mM Sucrose, 12.5 mM Tris-HCl, pH 7.5, 2.5 mM EDTA, pH 7.5). The cell lysate was centrifuged at 1300 × *g* for 5 min at 4 °C for two times, the supernatant was centrifuged at 17,000 × *g* for 15 min at 4 °C and the subsequent supernatant represented cytoplasmic fraction and the pellets represented crude mitochondria fraction.

### 4.10. Western Blotting

After treatment, cells were washed with ice-cold PBS and extracted in protein lysis buffer. Protein concentration was determined by the Bradford assay (Thermo Scientific, Waltham, MA, USA). Protein samples of cell lysate were mixed with an equal volume of 5× SDS sample buffer, boiled for 4 min, and then separated on 12% SDS-PAGE gels. After electrophoresis, proteins were transferred to polyvinylidene difluoride membranes. The membranes were blocked in 5% non-fat dry milk for 1 h, washed three times, and incubated with specific primary antibodies in Tris-buffered saline containing Tween-20 (TBST) overnight at 4 °C. Primary antibody was removed by washing the membranes three times in TBST, and incubated for 1 h with horseradish peroxidase-conjugated secondary antibody (1:1000–1:2000). Following three times of washing in TBST, immunopositive bands were visualized with the ECL kit (Amersham, Arlington Heights, IL, USA) according to the manufacturer’s instructions.

### 4.11. Ovarian Cancer Cell Xenograft Models

All experimental protocols and animal handling procedures were in accordance with the National Institutes of Health (NIH, Bethesda, MD, USA) guidelines for the use of experimental animals and the experimental protocols were approved by the Institutional Animal Care and Use Committee of the Fourth Military Medical University (Xi’an, China). SKOV3 cells (5 × 10^6^) were injected into BALB/c nude mice (6 weeks old). Mice were randomized and assigned to treatment and control group (40 mice enrolled in each group) and intraperitoneally injected every two days with PBS, 1.5 mg/kg fenretinide, 1.5 mg/kg selenite and combination of 1.5 mg/kg fenretinide and 1.5 mg/kg selenite, respectively. Single or dual treatment was started on 5 days after tumor cell implantation (1 mm^3^ tumor volume). Tumor diameters were measured every 5 days, the length (*L*) and the width (*W*) of the tumors were measured using a slide caliper (Guanglu, Guilin, China) and the volume (*V*) of each tumor was calculated as follows: *V* = *L* × *W*^2^/2. The experiment was terminated on day 50, and the tumors were weighed.

### 4.12. Statistical Analysis

All data were shown as mean ± standard deviation (s.d.) of three individual experiments performed in triplicate; significance was assessed using Student’s *t*-test. *p* < 0.05 was considered statistically significant.

## 5. Conclusions

In conclusion, Fenretinide and selenite combination treatment was demonstrated to suppress tumor growth *in vitro* and *in vivo*, this drug combination has been thus found to have an enhanced anti-tumor effect on ovarian cancers cells.

## Supplementary Information
