# Supplementary material for: Combination of Fenretinide and Selenite Inhibits Proliferation and Induces Apoptosis in Ovarian Cancer Cells
Source: Int J Mol Sci. 2013 Nov 4;14(11):21790–804. doi: 10.3390/ijms141121790 (PMC3856035; doi:10.3390/ijms141121790)
Supplement: Supplementary file 1 [file ijms-14-21790-s001.pdf]

Supplementary Information

Figure S1. The structure of fenretinide.

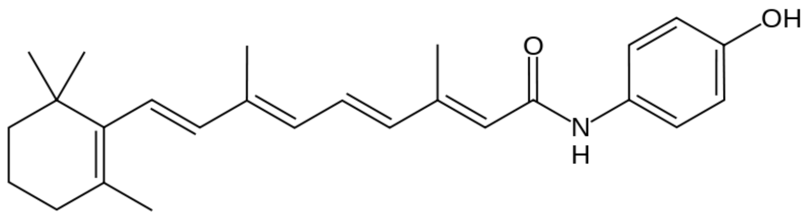

© 2013 by the authors; licensee MDPI, Basel, Switzerland. This article is an open access article distributed under the terms and conditions of the Creative Commons Attribution license (http://creativecommons.org/licenses/by/3.0/).
